# The Effect of Using a Smartphone App on Oral Hygiene and Brushing Training During Fixed Orthodontic Therapy: A Randomized Clinical Trial

**DOI:** 10.3390/diagnostics15182380

**Published:** 2025-09-18

**Authors:** Seda Sağoğlu, Mücahid Yıldırım

**Affiliations:** Department of Orthodontics, Faculty of Dentistry, Necmettin Erbakan University, 42090 Konya, Turkey; ssagoglu@erbakan.edu.tr

**Keywords:** fixed orthodontic appliances, oral hygiene motivation, oral hygiene, mobile application

## Abstract

**Objective:** This study aimed to study the effectiveness of a smartphone application compared to traditional verbal motivation in improving oral hygiene among fixed orthodontic patients. **Methods:** Sixty patients were categorized by oral hygiene status using the simplified oral hygiene index (OHI-S) and randomly assigned to either the Dentabuddy group (smartphone application) or the assistant-based training (ABT) group (conventional oral hygiene motivation). Gingival index (GI), plaque index (PI), and gingival bleeding index (GBI) values were recorded at baseline, one month, and three months. Toothbrushing technique was assessed at the three-month follow-up. **Results:** After three months, the Dentabuddy group exhibited significant GI reductions in participants with fair and poor oral hygiene, whereas the ABT group improved only in those with poor hygiene (*p* < 0.05). PI values decreased significantly in both groups, except in the ABT group with good and fair hygiene. GBI values improved in both groups, except in the ABT group with fair and poor hygiene (*p* < 0.05). Toothbrushing demonstrations showed superior technique in the Dentabuddy group (*p* < 0.05). **Conclusions:** The Dentabuddy application positively influenced oral hygiene, particularly in individuals with fair and poor hygiene, compared to ABT. This study underscores the potential of smartphone applications in enhancing periodontal health outcomes beyond traditional oral hygiene methods in orthodontic patients with fair or poor hygiene.

## 1. Introduction

Maintaining optimal oral hygiene represents an ongoing challenge for patients undergoing orthodontic treatment [1]. Plaque accumulation and gingivitis are frequent complications associated with orthodontic therapy, which, if not managed effectively, may lead to further oral health issues [2]. Further, motivation to maintain oral hygiene is crucial for preventing such side effects [3]. Fixed orthodontic treatments are most commonly applied in adolescent patients. In this group, age-related factors such as limited manual dexterity and low motivation to maintain oral hygiene are frequently observed. Moreover, the visibility of orthodontic appliances may lead to esthetic concerns, decreased self-confidence, and discomfort in interpersonal relationships; consequently, these factors can negatively affect treatment compliance and overall participation [3,4]. Orthodontists play a key role in fostering good oral hygiene through effective, trust-based communication with patients, which can contribute to a positive treatment experience and increase patient engagement [1]. Studies demonstrate that clear communication between patients and providers is integral to successful orthodontic outcomes [5].

In recent years, mobile technology has become increasingly embedded in daily life, altering how individuals interact with each other and their environments [6]. Today, two-thirds of the global population owns a smartphone, and the use of app-based technology to support both healthcare professionals and patients has expanded [7,8]. This growth in digital technology has positively influenced health behaviors. Smartphone applications aimed at monitoring oral health predominantly focus on enhancing oral hygiene practices [2,4]. Research indicates that oral hygiene instructions alone are often insufficient to produce significant improvements in terms of dental plaque control; such instructions prove more effective when supplemented with written explanations and visual aids [9]. Patient adherence is a vital component of oral hygiene, and providing reminders through video-supported smartphone apps or Short Message Service notifications can substantially improve compliance, helping reduce both plaque accumulation and gingivitis [10].

A review of the literature revealed that no study has compared changes in the oral hygiene levels of patients classified according to their oral hygiene status or used a smartphone application for such an analysis. The aim of this study was to compare changes in the gingival index (GI), plaque index (PI), and gingival bleeding index (GBI) values of individuals undergoing fixed orthodontic treatment. These patients had been classified into two groups based on their oral hygiene levels: those using the “Dentabuddy” application—designed to improve oral hygiene—and those receiving assistant-based toothbrushing training.

## 2. Material and Methods

Approval for this study was obtained from the Necmettin Erbakan University Faculty of Dentistry Non-Drug and Non-Medical Device Research Ethics Committee on 22 June 2023 (#2023/319). Further, informed consent was acquired from both the participants and their parents. Patients and/or parents were not involved in the design, conduct, reporting, or dissemination plans of this trial. This study was registered on ClinicalTrial.gov with TRN: NCT06865625 on 3 June 2025 (retrospectively registered) with no deviation from the original design.

### 2.1. Sample Size Calculation

The sample size was determined through power analysis using the G*Power 3.1 software (Franz Faul, Universität Kiel, Kiel, Germany) according to prior research [4]. With an effect size of 0.25, an α value of 0.05, and a standard deviation of 0.4 for the plaque index, the analysis indicated a sample size of 20 participants per group to achieve 80% power.

### 2.2. Inclusion/Exclusion Criteria

Patients aged 12–18 years with permanent dentition were included if they met the following criteria: (1) no missing teeth, (2) no systemic diseases or medications, (3) complete permanent dentition, (4) ability to maintain personal oral hygiene, and (5) ownership of an Android smartphone.

Exclusion criteria comprised ongoing dental treatments likely to influence oral hygiene or periodontal health, in addition to the presence of a smoking habit.

### 2.3. Participants and Randomization

This study was conducted between on patients who had been undergoing fixed orthodontic treatment for at least six months at the Department of Orthodontics, Necmettin Erbakan University Faculty of Dentistry (Table 1). All participants were examined by the first qualified researcher (SS) using an oral mirror and a periodontal probe. The assessment was repeated by the second qualified researcher (MY) two hours after the initial evaluation on the same day. The intraclass correlation coefficient (ICC) for gingival, plaque, and bleeding index measurements, as well as for the researchers, was determined to be 94.6%.

**Figure 1:**
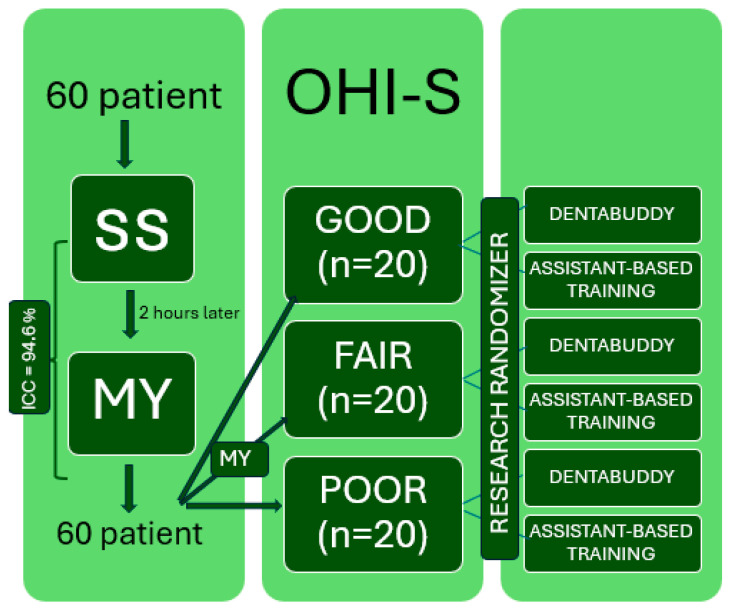
Study flowchart illustrating patient classification and randomization process.

Researcher MY also obtained simplified oral hygiene index (OHI-S) scores following Greene and Vermillion’s criteria, evaluating six tooth surfaces for debris and calculus to calculate each patient’s individual OHI-S score. Based on these scores, participants were categorized as having good (0.0–1.2), fair (1.3–3.0), or poor (3.1–6.0) oral hygiene [11]. A total of 20 participants from each category were randomly assigned to either the assistant-based training (ABT) or Dentabuddy group using the Research Randomizer website (https://www.randomizer.org; Figure 1). Following these procedures, all patients were examined at the 1- and 3-month follow-up visits, and their oral hygiene scores (GI, PI, and GBI) were recorded. All evaluations were conducted by the first qualified examiner (SS), who was blinded to the group allocation.

### 2.4. Study Design

All participants received routine periodontal treatment (Phase I) and oral hygiene training in the modified Bass technique, with assistant guidance using a model, before starting study.

**Figure 2:**
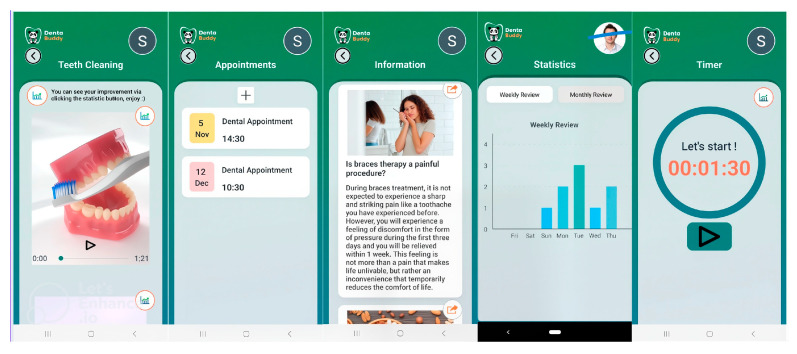
Screenshots of the Dentabuddy mobile application illustrating its core modules and user interface. The application includes the following sections: “Teeth Cleaning” (educational videos), “Appointments” (tracking past visits), “Information” (frequently asked questions), “Statistics” (monitoring brushing frequency), and “Timer” (tracking brushing duration). The image depicting toothache and sensitive teeth, utilized in our study, was obtained from Shutterstock, a publicly accessible stock photography platform. The visual material was sourced under Shutterstock’s user license agreement, which permits its use in academic contexts within the scope of licensed stock imagery (https://shutterstock.com/image-photo/toothache-pain-sensitive-teeth-woman-brushing-260nw-2191139955.jpg, accessed on 13 September 2025).

**Figure 3:**
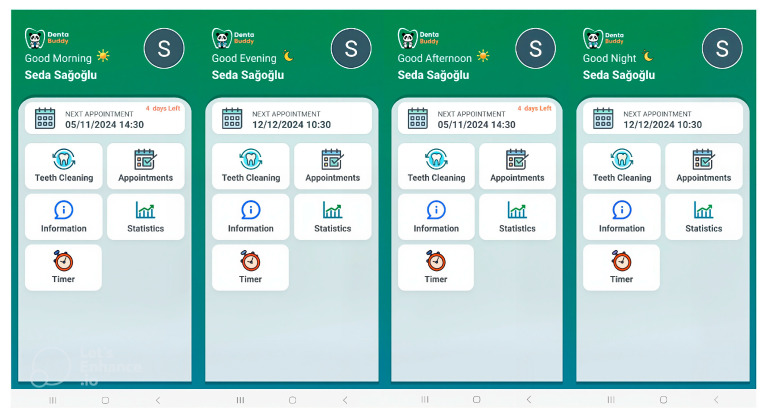
Screenshots of the time interval features of the Dentabuddy mobile application. Brushing sessions are logged according to specific time periods of the day: “Good morning” (04:00–12:00), “Good afternoon” (12:00–16:00), “Good evening” (16:00–20:00), and “Good night” (20:00–04:00).

The Dentabuddy application, which is freely available and publicly accessible on the Google Play Store, provides a user-friendly interface that allows login via Google, Twitter, or Facebook accounts. Upon login, users are presented with a home screen where upcoming appointment dates can be viewed, and from which different sections of the app can be accessed. The application includes instructional videos on toothbrushing, an appointment log, an appointment log, information, statistics, and a timer. Videos in the “Teeth Cleaning” section are approximately two minutes long, and brushing times are logged under set time periods: “Good morning (04:00–12:00),” “Good afternoon (12:00–16:00),” “Good evening (16:00–20:00),” and “Good night (20:00–04:00)” (Figure 2 and Figure 3). The instructional videos were prepared based on the steps of the modified Bass technique.

Participants in the Dentabuddy group downloaded and used the app under the guidance of the researcher MY, while those in the ABT group were instructed to brush three times daily for two minutes per session and log brushing frequency on a chart. Initial oral hygiene motivation, provided at the onset of the treatment, was reinforced for both groups at the beginning of the study. Patients in the ABT group were instructed to brush their teeth three times a day—morning, evening, and noon—with each session lasting two minutes. In the Dentabuddy group, participants were instructed to follow the brushing videos within the application, ensuring compliance with the different time periods while brushing. Brushing data obtained from the Dentabuddy application were collected through a dedicated email account established under the supervision of MY. To standardize the data collection process, participants were instructed to submit screenshots of the “Statistics” section of the application, displaying their weekly and monthly brushing counts, via their personal mobile phones to the designated email address.

### 2.5. Clinical Outcome Measures

The PI and GI, as described by Löe and Silness, were employed for all teeth [12]. In addition, the GBI values were assessed by gently gliding a periodontal probe along the gingival groove. If bleeding was observed within 10 s, it was recorded as a positive result; the number of positive sites were tallied, and the bleeding percentage was calculated by dividing the number of positive sites by the total number of tooth sites [13]. The GI, PI, and GBI values for the participants were recorded using a periodontal probe at the start of the study (T0) and during the one-month (T1) and three-month (T2) follow-up visits (Table 2 and Table 3).

**Figure 4:**
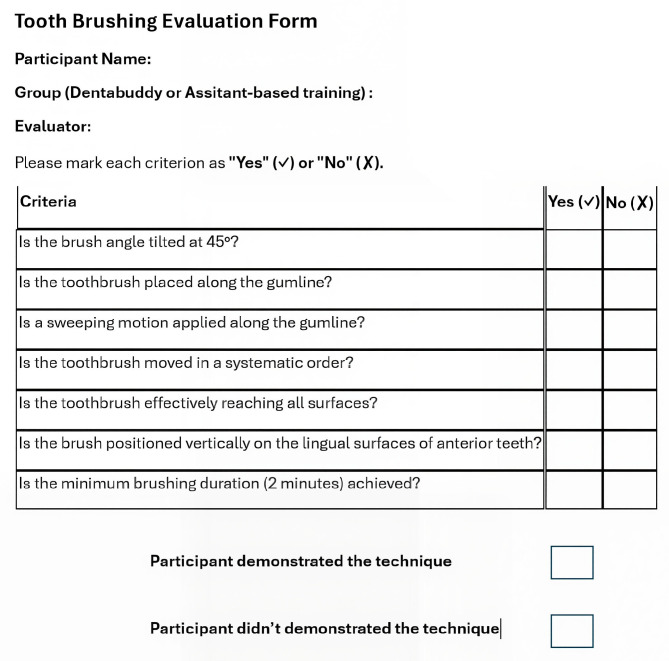
Toothbrushing evaluation form.

At the end of the three-month follow-up, all participants were asked to demonstrate their brushing technique on a bracket model. Their performance was evaluated using a structured checklist (Figure 4), which included criteria such as correct toothbrush positioning at a 45° angle to the gingival margin, the use of gentle vibratory and circular motions, adequate coverage of all tooth surfaces (buccal, lingual, and occlusal), appropriate brushing duration, and systematic movement across the dental arches. Participants who fulfilled at least four criteria on the evaluation form were marked as having “demonstrated the proper technique,” while those who met three or fewer criteria were marked as “did not demonstrate the proper technique.” by the first examiner (SS).

### 2.6. Statistical Analysis

The data were analyzed using SPSS version 25.0 (IBM Corp., Armonk, NY, USA). Descriptive statistics are presented as mean ± SD for continuous data and as frequency (percentage) for categorical data. The normality of the index values and brushing frequency was determined using the Kolmogorov–Smirnov test, and it was confirmed that the data follow a normal distribution. Comparisons between the two groups were conducted using Student’s *t*-test, while one-way ANOVA was used for comparisons across multiple groups; Tukey’s HSD was employed as the post hoc test for significant outcomes. Further, a repeated-measures analysis of variance was used to evaluate changes in the index measurements over time, and a chi-squared analysis was utilized to determine associations between categorical variables. Finally, a *p*-value of less than 0.05 indicated a statistical significance of 5% for a type I error rate.

## 3. Results

**Figure 5:**
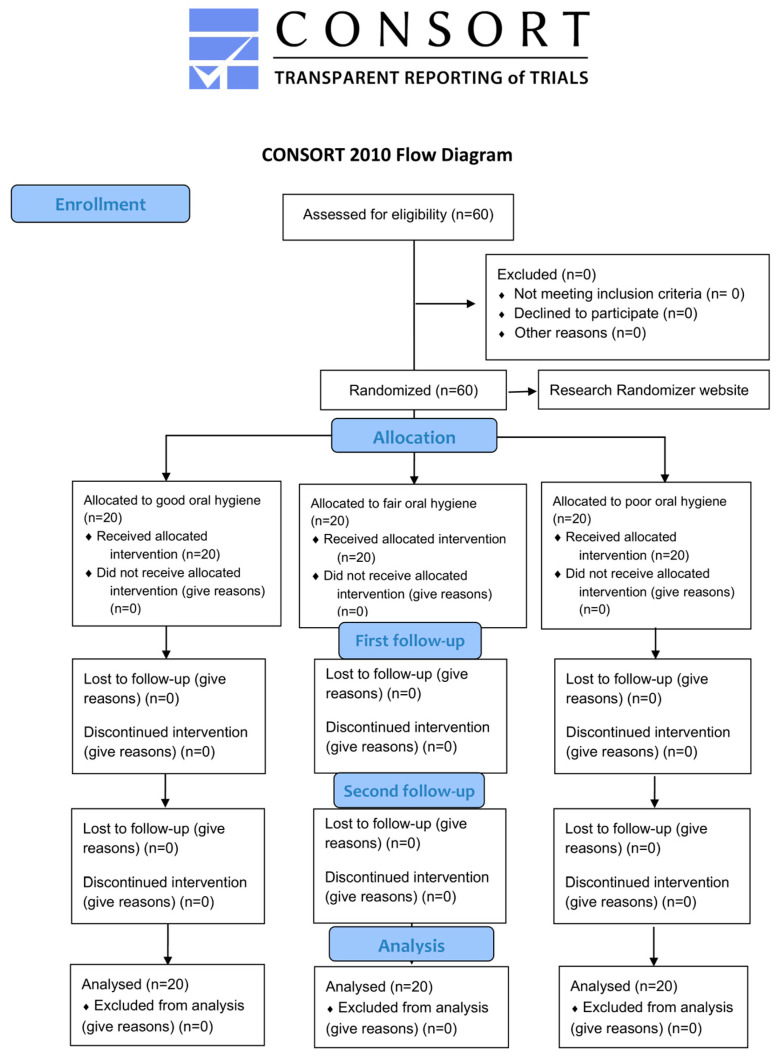
CONSORT diagram.

Patients were recruited between August 2023 and November 2023. A total of 60 patients were assessed for eligibility, all of whom met the inclusion criteria and were subsequently randomized equally into six groups (*n* = 10 per group). All participants received the allocated intervention. The patient recruitment process is illustrated in Figure 5.

For patients with good oral hygiene, the PI was higher in the ABT group at T0 (*p* = 0.030), with no differences observed in the GI or GBI. At T1, the GI was higher in the Dentabuddy group, while the GBI was higher in the ABT group (*p* = 0.002 and *p* < 0.001, respectively); no significant difference was found in PI values (*p* > 0.05). At T2, all parameters were reduced in the Dentabuddy group (*p* < 0.001), and no significant differences were observed in the parameter measurements of either group between T0 and T1 (*p* > 0.05). Moreover, the change in the GI between T0 and T2 was not significant in either group. While the PI and GBI decreased in the Dentabuddy group, the GBI increased in the ABT group (*p* < 0.001; Table 4 and Table 5).

For patients with fair oral hygiene, no differences were found in the index values at T0 (*p* > 0.05). At T1 and T2, the Dentabuddy group presented reduced values across all parameters (*p* < 0.05), and no significant differences were found in the GI, PI, and GBI for either group between T0 and T1 (*p* > 0.05). Between T0 and T2, a decrease was observed in all parameters in the Dentabuddy group (*p* < 0.05), with no significant changes noted for the ABT group (*p* > 0.05; Table 4 and Table 5).

For patients with poor oral hygiene, no differences were found in the index values at T0 (*p* > 0.05). At T1, the GI and GBI values were reduced in the Dentabuddy group (*p* = 0.015 and *p* = 0.006, respectively); while the PI values were also reduced in the Dentabuddy group, the difference was not significant (*p* > 0.05). At T2, all index values were lower in the Dentabuddy group (*p* < 0.05). Between T0 and T1, the GI and PI were reduced in the ABT group, while the GBI was reduced in the Dentabuddy group (*p* < 0.05). From T0 to T2, all the index values decreased in the Dentabuddy group (*p* = 0.002), while the GI and PI values decreased in the ABT group (*p* = 0.012 and *p* = 0.004, respectively). Finally, no significant changes were seen in the GBI values in the ABT group (*p* > 0.05; Table 4 and Table 5).

In addition to *p*-values, effect sizes and their corresponding confidence intervals are presented in Table 6 to provide a clearer indication of the magnitude and precision of the observed differences.

In addition, no significant differences were found in the number of brushing sessions reported at T1 and T2 in the Dentabuddy and ABT groups according to oral hygiene levels (*p* = 0.724 and *p* = 0.825, respectively; Table 7).

Last, the brushing demonstration rate was significantly higher for the Dentabuddy group (*p* = 0.038), with 70% of participants in the Dentabuddy group demonstrating proper brushing technique, as compared to 40% in the ABT group (Table 8).

## 4. Harms

No patients were harmed during the study.

## 5. Discussion

In this study, changes in the GI, PI, and GBI values were compared among individuals undergoing fixed orthodontic treatment, who were classified based on their oral hygiene levels. The comparison was made between those who used the Dentabuddy application, which is designed to enhance oral hygiene, and those who received assistant-based toothbrushing training.

Approaches targeting young people, particularly those that incorporate playful language and tools such as smartphones and visual materials, are frequently employed in educational settings [2]. Smartphone applications tailored to orthodontic patients present an innovative platform for delivering oral hygiene education [2]. Prior research involving oral health interventions for adolescents through media-based and messaging apps—including videos, selfies, text messages, and face-to-face guidance—has demonstrated improvements in participants’ oral health-related knowledge, attitudes, and behaviors [2]. Thus, a sample group of 12–18-year-olds was selected for this study.

Previous studies indicate that patients who are new to fixed orthodontic treatment need to acquire and adapt to new manual skills to maintain optimal oral hygiene with braces, especially during the initial months [4,13]. As the treatment progresses, patient motivation often diminishes, which can lead to a degradation of oral hygiene practices. Hence, motivational strategies are essential to sustain patient compliance over the course of the treatment [14]. For this reason, this study included patients who had been undergoing fixed orthodontic treatment for over six months, possessed adequate brushing skills, and were likely experiencing decreased motivation due to the duration of their treatment. In a related study, Ajayi and Azodo assessed the oral hygiene status of patients undergoing active orthodontic treatment using the OHI-S [15]. Similarly, in this study, patients were categorized into three groups—good, fair, and poor oral hygiene—based on OHI-S scores, which enabled an analysis of the effectiveness of a smartphone application as an oral hygiene motivational tool for patients with varying levels of oral hygiene.

There have been numerous attempts to formulate effective strategies to improve adherence to oral hygiene instructions in orthodontic patients, and interventions such as pre-treatment oral hygiene education programs and illustrated brochures have shown positive results [9]. Given technological advancements, methods involving smartphone applications and social media reminders have proven more effective than traditional approaches. Informative videos created by dentists or orthodontists and published on social media or video platforms (e.g., YouTube), along with media-based brochures and smartphone health applications—especially in general dentistry and orthodontics—have been utilized to enhance oral health awareness and motivation [16,17,18]. Although smartphone applications have become widely accessible due to internet proliferation, orthodontic app development has not advanced as rapidly as other categories. Between 2013 and 2017, the number of orthodontic apps on major platforms, such as the App Store^®^ and Google Play^®^, increased from 19 to 354 [19]. The recent evaluation of orthodontic apps by Siddiqui using the mobile app rating scale (MARS) and behavior change techniques (BCTs) highlighted the limited availability of high-quality apps for patients, thus underscoring the need for applications with robust behavior modification features [20]. Apps that monitor oral hygiene offer practical benefits to both the patients and the institutions providing care due to their low cost and ease of implementation [4,21,22].

Orthodontic patients, predominantly belonging to younger age groups, often struggle to focus on the long-term benefits of treatment and tend instead to prioritize immediate esthetic concerns and discomfort. Consequently, researchers have long sought effective strategies to enhance patients’ adherence to oral hygiene instructions. Educational programs and illustrated brochures delivered prior to the initiation of treatment have been shown to improve compliance. In addition, the use of smartphone reminders or the delivery of weekly text messages has been associated with improved oral hygiene practices and a reduction in patient-reported pain [9].

Given the critical importance of compliance in orthodontic treatment, the development of novel approaches to support adherence remains an active area of research. With the increasing prevalence of smartphone use, mobile applications designed to promote patient education and compliance have attracted growing attention. Indeed, previous studies have demonstrated that such applications can effectively improve oral hygiene among individuals with fixed orthodontic appliances [2,4,9].

Many studies in the literature assess the impact of such applications on the oral health behaviors of patients undergoing fixed orthodontic treatment. Zotti et al. conducted a study with individuals similar to those in our good oral hygiene group (PI scores 0–1) and compared the effects of a WhatsApp-based chat room, named “Brush Game,” with a standard oral hygiene protocol in patients undergoing fixed orthodontic treatment [2]. In this study, each patient was evaluated every three months for one year, and GI and PI scores were recorded at each visit. At the three-month follow-up, no difference was observed in the GI scores of the chat room group, whereas significant increases were found in the ABT group. The findings obtained for the Dentabuddy group are consistent with this study. However, the significant increase in PI values recorded in both groups at the end of the first three months does not correspond with the findings of the present study. The authors attributed the absence of differences between baseline and the third month to the insufficient development of oral hygiene skills during the early stages of orthodontic treatment. In our study, however, participants continued their existing brushing habits while being supported by the application. In another study reporting similar PI scores, Alkadhi et al. compared video-supported smartphone applications with verbal oral hygiene motivation in patients undergoing orthodontic treatment and reported reductions in both GI and PI scores in the application group after four weeks [4]. The decrease in PI scores observed in this study parallels these findings, and the application used was reported to provide significant improvements in oral hygiene outcomes.

Deleuse et al. investigated patients aged 12–17 years with fair oral hygiene (PI scores 1–2), comparable to our sample, and compared the effects of an interactive electric toothbrush integrated with an application to those of a non-interactive electric toothbrush [1]. In this study, both groups exhibited significant decreases in GI scores, consistent with the data from the Dentabuddy group. Deleuse et al. [1] further reported that between the sixth and twelfth weeks, PI scores decreased only in the interactive toothbrush group, while an increase was observed in the non-interactive group. In contrast, in our study, decreases were observed in both groups. The intergroup differences reported at the 12th week in their study are consistent with our T2 findings. Scheerman et al. examined the effects of the “WhiteTeeth” application on oral health behaviors and oral hygiene in orthodontic patients [23]. In the application group, reductions in PI and GI values were observed at the sixth week, and this effect was reported to persist at the twelfth week. Similarly, Dos Santos et al. reported decreases in PI and GBI scores at a three-month follow-up in the group using a mobile application, while Davoodi et al. found reductions in PI and GI scores at the third month following the use of a mobile application developed for oral hygiene motivation [24]. Shafaee et al., even with a short one-month follow-up, reported significant improvements in both PI and GI values in the application group [18]. In this study as well, the reductions observed across all oral parameters in the Dentabuddy group at the three-month follow-up are consistent with these findings. Taken together, these results suggest that digital applications can be effective in supporting plaque control and gingival health, particularly in the early stages of treatment, and that continued use of such applications may contribute to the sustainability of these positive outcomes.

Further, the authors found no differences between groups in terms of the frequency of toothbrushing across any time period, which aligns with our study results [23]. The findings indicate that toothbrushing frequency alone is not sufficient for maintaining periodontal health, and that the primary effect is achieved through the correct application of brushing technique. In this context, mobile applications emerge not only as tools that enhance behavioral motivation, but also as digital interventions with the potential to standardize and improve toothbrushing technique.

Recent studies evaluating the oral hygiene and hygiene habits of patients undergoing orthodontic treatment indicate that smartphone applications specifically designed for oral hygiene education can effectively help reduce plaque accumulation and minimize the risk of gingivitis during orthodontic treatment [18,25]. The benefits of using videos to enhance oral hygiene motivation—including easy accessibility, clear information delivery, and the enabling of self-paced learning—combined with integration into a smartphone application, allow patients to more actively engage in the learning process. This integration in the Dentabuddy application increased patients’ interest in using the app and, consistent with the literature, led to improved oral hygiene parameters compared to traditional motivation methods.

This study focused on toothbrushing and related oral hygiene behaviors. Dentabuddy was developed to address the needs of patients undergoing orthodontic treatment who present with insufficient brushing duration and deficiencies in toothbrushing technique. A three-month follow-up period was selected as it provides an appropriate timeframe to observe meaningful behavioral changes and the stabilization of these habits. Scheerman et al. demonstrated that a three-month follow-up is sufficient and effective for evaluating oral hygiene compliance in patients with fixed orthodontic appliances [23]. The findings of this study demonstrate that the application provided measurable short-term improvements in oral hygiene outcomes. Considering the specific challenges associated with the examined age group, tailoring digital applications to their needs may enhance the duration, frequency, and quality of use. Furthermore, ensuring the long-term sustainability of these applications could be effective in modifying oral health behaviors during adolescence and supporting their maintenance throughout life.

Although digital interventions can enhance adherence to oral hygiene practices, biological approaches such as probiotic supplementation have also been investigated for their potential benefits in controlling gingival inflammation [26]. Future studies may consider evaluating the complementary effects of integrating digital strategies with biological interventions, which could contribute to further improvements in periodontal outcomes during orthodontic therapy.

## 6. Limitations

The follow-up period in our study was set to three months. While this duration is sufficient for establishing oral hygiene habits, a longer follow-up may be necessary to assess whether these habits are being sustained in patients’ daily routines. Another limitation of this study is its relatively small sample size, which may affect the generalizability of the findings. Additionally, the lack of blinding for the outcome evaluator is acknowledged as a limitation; however, objective measurement methods were employed to minimize potential bias. Future studies should consider employing larger sample sizes and blinded evaluations to enhance the reliability and applicability of their results.

In future studies, it is anticipated that longitudinal follow-up of individuals until the completion of orthodontic treatment, along with the inclusion of additional parameters such as the development of white spot lesions and bracket failures, will provide novel and meaningful contributions to the literature.

## 7. Conclusions

The age groups included in this study demonstrated high adherence to digital strategies for promoting oral hygiene. Notably, during treatment, providing hygiene motivation to patients with poor oral hygiene resulted in measurable improvements. Moreover, individuals with fair and poor oral hygiene achieved more hygienic results through digital monitoring. The use of the application facilitated the learning of proper brushing techniques.

It would be beneficial for future studies to compare the effects of such applications across different age groups. Moreover, integrating these applications into treatment programs that allow clinicians to oversee patients’ progress or incorporating artificial intelligence-assisted features may enhance their usability and effectiveness.

## Figures and Tables

**Table 1:** Research subjects’ distribution according to OHI-S, age and gender.

Group	Age (Years)	OHI-S	Female	Male
		Good	5	5
Dentabuddy	16.56 ± 1.49	Fair	5	5
		Poor	5	5
		Good	5	5
Assistant-based training	16.87 ± 1.55	Fair	5	5
		Poor	5	5

**Table 2:** Gingival index scores and criteria.

Score	Criteria
0	Healthy gingiva—absence of inflammation, bleeding, or swelling
1	Mild inflammation—slight change in color, slight edema. No bleeding on probing
2	Moderate inflammation—redness, edema, and glazing. Bleeding on probing
3	Severe inflammation—marked redness and edema, ulceration. Tendency to spontaneous bleeding

**Table 3:** Plaque index scores and criteria.

Score	Criteria
0	No plaque in the gingival area
1	Slight deposit of plaque at gingival margin
2	Moderate deposit of plaque covering less than half of the surface
3	Important deposit of plaque covering more than half of the surface

**Table 4:** Index values by oral hygiene and study groups.

			T0		T1		T2	
Oral Hygiene	Index Values	Groups	Mean ± SD	*p*	Mean ± SD	*p*	Mean ± SD	*p*
**GOOD**	**Gingival index**	Dentabuddy	0.14 ± 0.04	0.072	0.21 ± 0.24	0.002 *	0.11 ± 0.02	<0.001 *
		Assistant-based training	0.17 ± 0.03		0.17 ± 0.03		0.17 ± 0.03	
	**Plaque index**	Dentabuddy	0.11 ± 0.04	0.030 *	0.16 ± 0.26	0.915	0.05 ± 0.02	<0.001 *
		Assistant-based training	0.14 ± 0.03		0.15 ± 0.03		0.15 ± 0.03	
	**Gingival bleeding index**	Dentabuddy	2.19 ± 0.91	0.152	1.15 ± 0.96	<0.001 *	0.52 ± 0.62	<0.001 *
		Assistant-based training	2.81 ±0.96		2.19 ± 0.91		4.03 ± 1.71	
**FAIR**	**Gingival index**	Dentabuddy	1.19 ± 0.14	0.702	0.97 ± 0.19	0.005 *	0.74 ± 0.52	0.015 *
		Assistant-based training	1.21 ± 0.10		1.20 ± 0.12		1.24 ± 0.14	
	**Plaque index**	Dentabuddy	1.09 ± 0.09	0.835	0.84 ± 0.19	0.007 *	0.65 ± 0.51	0.018 *
		Assistant-based training	1.10 ± 0.10		1.07 ± 0.13		1.12 ± 0.15	
	**Gingival bleeding index**	Dentabuddy	21.56 ± 2.66	0.955	12.94 ± 6.99	0.002 *	10.14 ± 8.79	0.002 *
		Assistant-based training	21.49 ± 2.69		21.80 ± 3.71		22.36 ± 4.19	
**POOR**	**Gingival index**	Dentabuddy	2.09 ± 0.11	0.691	1.75 ± 0.27	0.015 *	1.36 ± 0.52	0.004 *
		Assistant-based training	2.07 ± 0.07		2.00 ± 0.10		2.01 ± 0.09	
	**Plaque index**	Dentabuddy	2.09 ± 0.09	0.322	1.74 ± 0.29	0.063	1.33 ± 0.58	0.010 *
		Assistant-based training	2.05 ± 0.07		1.94 ± 0.09		1.93 ± 0.08	
	**Gingival bleeding index**	Dentabuddy	31.60 ± 8.83	0.985	20.84 ± 7.92	0.006 *	12.53 ± 7.63	<0.001 *
		Assistant-based training	31.67 ± 8.03		32.02 ± 8.27		31.91 ± 8.69	

T0, indicates baseline; T1, after 1 months; T2, after 3 months. *: *p* < 0.05, Student *t*-test.

**Table 5:** Index values by measurement periods.

			T0	T1	T2 ^+^	*p*
Oral Hygiene	Index Values	Groups	Mean ± SD	Mean ± SD	Mean ± SD	
**GOOD**	**Gingival index**	Dentabuddy	0.14 ± 0.04	0.21 ± 0.24	0.11 ± 0.02	0.173
		Assistant-based training	0.17 ± 0.03	0.17 ± 0.03	0.17 ± 0.03	0.938
	**Plaque index**	Dentabuddy	0.11 ± 0.04	0.16 ± 0.26	0.05 ± 0.02	<0.001 **
		Assistant-based training	0.14 ± 0.03	0.15 ± 0.03	0.15 ± 0.03	0.696
	**Gingival bleeding index**	Dentabuddy	2.19 ± 0.91	1.15 ± 0.96	0.52 ± 0.62	<0.001 **
		Assistant-based training	2.81 ±0.96	2.19 ± 0.91	4.03 ± 1.71	<0.001 **
**FAIR**	**Gingival index**	Dentabuddy	1.19 ± 0.14	0.97 ± 0.19	0.74 ± 0.52	0.016 **
		Assistant-based training	1.21 ± 0.10	1.20 ± 0.12	1.24 ± 0.14	0.384
	**Plaque index**	Dentabuddy	1.09 ± 0.09	0.84 ± 0.19	0.65 ± 0.51	0.018 **
		Assistant-based training	1.10 ± 0.10	1.07 ± 0.13	1.12 ± 0.15	0.505
	**Gingival bleeding index**	Dentabuddy	21.56 ± 2.66	12.94 ± 6.99	10.14 ± 8.79	0.002 **
		Assistant-based training	21.49 ± 2.69	21.80 ± 3.71	22.36 ± 4.19	0.216
**POOR**	**Gingival index**	Dentabuddy	2.09 ± 0.11	1.75 ± 0.27	1.36 ± 0.52	0.002 **
		Assistant-based training	2.07 ± 0.07	2.00 ± 0.10	2.01 ± 0.09	0.012 **
	**Plaque index**	Dentabuddy	2.09 ± 0.09	1.74 ± 0.29	1.33 ± 0.58	0.002 **
		Assistant-based training	2.05 ± 0.07	1.94 ± 0.09	1.93 ± 0.08	0.004 **
	**Gingival bleeding index**	Dentabuddy	31.60 ± 8.83	20.84 ± 7.92	12.53 ± 7.63	0.002 **
		Assistant-based training	31.67 ± 8.03	32.02 ± 8.27	31.91 ± 8.69	0.712

T0, indicates baseline; T1, after 1 months; T2, after 3 months. **: *p* < 0.05, Repeated Measures Analysis of Variance. ^+^: Measurement period demonstrating a significant difference in pairwise comparisons between time points.

**Table 6:** Group comparisons of oral hygiene parameters (GI, PI, GBI) with *p*-values, effect sizes, and 95% confidence intervals.

				Cohen’s D		Cohen’s D		Cohen’s D	*p*	Eta-Square
Oral Hygiene	Index Values	Groups	*p*	95% CI	*p*	95% CI	*p*	95% CI		90% CI
**GOOD**	**Gingival index**	Dentabuddy	0.072	0.854	0.002 *	−0.213	<0.001 *	2.058	0.173	0.196 (0.00–0.479)
		Kontrol		−1.839		−1.758		0.938–3.141	0.938	0.001 (0.00–0.032)
	**Plaque index**	Dentabuddy	0.030 *	1.055	0.915	−0.048	<0.001 *	3.687	<0.001 **	0.795 (0.454–0.871)
		Kontrol		0.101–1.983		−1.753		2.187–5.151	0.696	0.017 (0.00–0.258)
	**Gingival bleeding index**	Dentabuddy	0.152	0.668	<0.001 *	2.289	<0.001 *	2.726	<0.001 **	0.783 (0.429–0.863)
		Kontrol		−1.805		1.122–3.419		1.462–3.951	<0.001 **	0.499 (0.071–0.688)
**FAIR**	**Gingival index**	Dentabuddy	0.702	0.174	0.005 *	1.424	0.015 *	1.31	0.016 **	0.492 (0.066–0.683)
		Kontrol		−1.757		0.418–2.399		0.321–2.270	0.384	0.084 (0.00–0.374)
	**Plaque index**	Dentabuddy	0.835	0.095	0.007 *	1.368	0.018 *	1.249	0.018 **	0.480 (0.058–0.676)
		Kontrol		−1.754		0.371–2.336		0.269–2.201	0.505	0.051 (0.00–0.329)
	**Gingival bleeding index**	Dentabuddy	0.955	−0.026	0.002 *	1.583	0.002 *	1.776	0.002 **	0.664 (0.234–0.791)
		Kontrol		−1.753		0.551–2.582		0.710–2.808	0.216	0.164 (0.00–0.452)
**POOR**	**Gingival index**	Dentabuddy	0.691	−0.18	0.015 *	1.205	0.004 *	1.717	0.002 **	0.662 (0.232–0.789)
		Kontrol		−1.757		0.231–2.150		0.662–2.739	0.012 **	0.518 (0.085–0.700)
	**Plaque index**	Dentabuddy	0.322	−0.456	0.063	0.927	0.010 *	1.452	0.002 **	0.670 (0.242–0.793)
		Kontrol		−1.778		−1.853		0.442–2.432	0.004 **	0.617 (0.177–0.761)
	**Gingival bleeding index**	Dentabuddy	0.985	0.008	0.006 *	1.38	<0.001 *	2.37	0.002 **	0.829 (0.527–0.892)
		Kontrol		−1.753		0.381–2.349		1.186–3.517	0.712	0.015 (0.00–0.251)

*: *p* < 0.05, Student *t*-test. **: *p* < 0.05, Repeated Measures Analysis of Variance.

**Table 7:** Comparison of brushing frequency by study groups.

		Assistant-Based Training			Dentabuddy		
Number of Brushings	Oral Hygiene	Mean	SD	*p*	Mean	SD	*p*
T0–T1	Good	76.3	9.71	0.995	74.8	14.52	0.724
	Fair	76.1	11.85		80.4	16.38	
	Poor	75.8	12.71		78.5	16.28	
T1–T2	Good	138.9	19.27	0.849	139.9	25.22	0.825
	Fair	143.9	22.21		148.7	34.75	
	Poor	143.8	24.99		141.8	38.55	

**Table 8:** Distribution of toothbrushing demonstration status by study groups.

		Assistant-Based Training	Dentabuddy	
		*N* (%)	*N* (%)	*p*
Brushing demonstration	No	18 (60.0)	9 (30.0)	0.038 *
	Yes	12 (40.0)	21 (70.0)	

* *p* < 0.05, Chi-square analysis with Yates’ correction.

## Data Availability

The data presented in this study are available on request from the corresponding author due to privacy and ethical restrictions.
